# Perioperative electroencephalography in cardiac surgery with hypothermic circulatory arrest: a narrative review

**DOI:** 10.1093/icvts/ivac198

**Published:** 2022-07-29

**Authors:** William M McDevitt, Tanwir Gul, Timothy J Jones, Barnaby R Scholefield, Stefano Seri, Nigel E Drury

**Affiliations:** Department of Neurophysiology, Birmingham Children’s Hospital, Birmingham, UK; School of Biomedical Sciences, University of Birmingham, Birmingham, UK; Department of Paediatric Cardiac Surgery, Birmingham Children’s Hospital, Birmingham, UK; Department of Paediatric Cardiac Surgery, Birmingham Children’s Hospital, Birmingham, UK; Institute of Cardiovascular Sciences, University of Birmingham, Birmingham, UK; Institute of Inflammation and Ageing, University of Birmingham, Birmingham, UK; Paediatric Intensive Care Unit, Birmingham Children’s Hospital, Birmingham, UK; Department of Neurophysiology, Birmingham Children’s Hospital, Birmingham, UK; College of Health and Life Sciences, Aston University, Birmingham, UK; Department of Paediatric Cardiac Surgery, Birmingham Children’s Hospital, Birmingham, UK; Institute of Cardiovascular Sciences, University of Birmingham, Birmingham, UK

**Keywords:** Review, Paediatric cardiac surgery, Electroencephalography, Neuroprotection, Hypothermic circulatory arrest, Neurological injury

## Abstract

**OBJECTIVES:**

Cardiac surgery with hypothermic circulatory arrest (HCA) is associated with neurological morbidity of variable severity and electroencephalography (EEG) is a sensitive proxy measure of brain injury. We conducted a narrative review of the literature to evaluate the role of perioperative EEG monitoring in cardiac surgery involving HCA.

**METHODS:**

Medline, Embase, Central and LILACS databases were searched to identify studies utilizing perioperative EEG during surgery with HCA in all age groups, published since 1985 in any language. We aimed to compare EEG use with no use but due to the lack of comparative studies, we performed a narrative review of its utility. Two or more reviewers independently screened studies for eligibility and extracted data.

**RESULTS:**

Fourty single-centre studies with a total of 3287 patients undergoing surgery were identified. Most were observational cohort studies (34, 85%) with only 1 directly comparing EEG use with no use. EEG continuity (18, 45%), seizures (15, 38%) and electrocerebral inactivity prior to circulatory arrest (15, 38%) were used to detect, monitor, prevent and prognose neurological injury. Neurological dysfunction was reported in almost all studies and occurred in 0–21% of patients. However, the heterogeneity of reported clinical and EEG outcome measures prevented meta-analysis.

**CONCLUSIONS:**

EEG is used to detect cortical ischaemia and seizures and predict neurological abnormalities and may guide intraoperative cerebral protection. However, there is a lack of comparative data demonstrating the benefit of perioperative EEG monitoring. Use of a standardized methodology for performing EEG and reporting outcome metrics would facilitate the conduct of high-quality clinical trials.

## INTRODUCTION

Hypothermia remains an essential technique to protect the brain during cardiac surgical procedures which require circulatory arrest [1]. As core temperature decreases, cerebral metabolism is reduced, thereby offering a window of neuroprotection, with lower core temperatures targeted for more complex and extensive repairs that require prolonged arrest. Despite the use of hypothermia in conjunction with cerebral perfusion techniques, neuromonitoring and neuroprotective drug regimes, new postoperative neurological deficits can still occur in both adults and children undergoing cardiac surgery, and the reason for this is not completely understood [2, 3].

Electroencephalography (EEG) records the summated postsynaptic potentials of neural tissue from electrodes placed on the scalp [4]. This activity is classified as normal or abnormal based on its location, morphology and amplitude relative to the age of the patient. EEG can be used to detect seizures and signs of ischaemia and guide the depth of anaesthesia and hypothermia [5, 6]. In spite of its wide availability in clinical centres, perioperative EEG monitoring is not routinely used during cardiac surgery with hypothermic circulatory arrest (HCA). A recent survey on its use during aortic arch surgery suggested that it is only performed routinely in ∼17% of European centres [7]. Guidelines from the American Society of Neurophysiological Monitoring consider EEG in cardiac surgery with cardiopulmonary bypass (CPB) to be a practice option rather than a standard of care, as there are no standards for patient management or established role in improving outcomes [8]. We therefore conducted a narrative review of studies reporting EEG technique and/or outcomes of perioperative EEG monitoring in children and adults undergoing cardiac surgery with HCA to evaluate whether there was consistency in methodology, synthesize the current evidence base and identify any impact on postoperative outcomes.

## MATERIALS AND METHODS

This review was conducted with reference to the Cochrane handbook for reviews of interventions [9] and reported in accordance with the PRISMA statement [10]. All eligibility criteria, search terms and data items were prespecified, and the review was prospectively registered on PROSPERO (CRD42021247700) (https://www.crd.york.ac.uk/prospero).

### Study eligibility

We included patients of all ages undergoing cardiac surgery with HCA. We identified studies assessing perioperative EEG monitoring (processed or unprocessed) and whether the study compared the use of EEG monitoring with no EEG monitoring. Outcomes of interest were neurological status, which encompassed any observer-reported outcome indicating neurological dysfunction and perioperative EEG technique. We included all randomized controlled trials (RCTs), non-randomized trials, prospective and retrospective observational cohorts, case series and cross-sectional studies published in any language since 1985 so that retrieved articles better reflected current perioperative management.

Cardiac surgery was defined as any therapeutic clinical procedure performed on the heart or great vessels and HCA as cessation of the circulation following systemic cooling via CPB. EEG was defined as the subjective or quantitative interpretation of cortical activity recorded from at least 2 electrodes placed on the scalp.

Secondary publications, sub-studies or long-term outcomes of previously reported studies were excluded unless the results were specifically related to the utility of EEG monitoring or reported additional neurological outcome measures. Studies published only as a conference abstract, or for which all options to obtain the full text were exhausted, were excluded due to insufficient data.

### Search strategy

We searched international primary research databases (Medline, Embase, Central, LILACS) from 1 January 1985 to 13 May 2022 and reference lists of relevant articles, systematic reviews and meta-analyses to identify all eligible studies. The search terms used were comprehensive and adapted for each database, with database-specific filters to identify the population and intervention of interest (see Supplementary material).

### Study selection and data extraction

Title and abstracts and then full-text publications of all identified articles were screened independently by 2 reviewers (William M. McDevitt and Tanwir Gul) to generate a database of included studies. Data were extracted independently by 2 reviewers (2 of William M. McDevitt, Tanwir Gul and Nigel E. Drury) from the full text and any published protocols or Supplementary material; a full list of data items and descriptors is available in the Supplementary material. Non-English articles were translated and any disagreements on study selection or data extraction were resolved by consensus.

### Statistical analysis

Continuous data were expressed as median with interquartile range, mean with standard deviation or range. Categorical data were expressed as counts and percentages where relevant. We did not plan to perform a meta-analysis or analyse sensitivity and homogeneity because we did not anticipate retrieving homogenous studies relatively resistant to bias. In the event of no or limited studies comparing EEG monitoring with no EEG monitoring, we planned to undertake a narrative review of studies describing perioperative EEG monitoring.

## RESULTS

**Figure 1:**
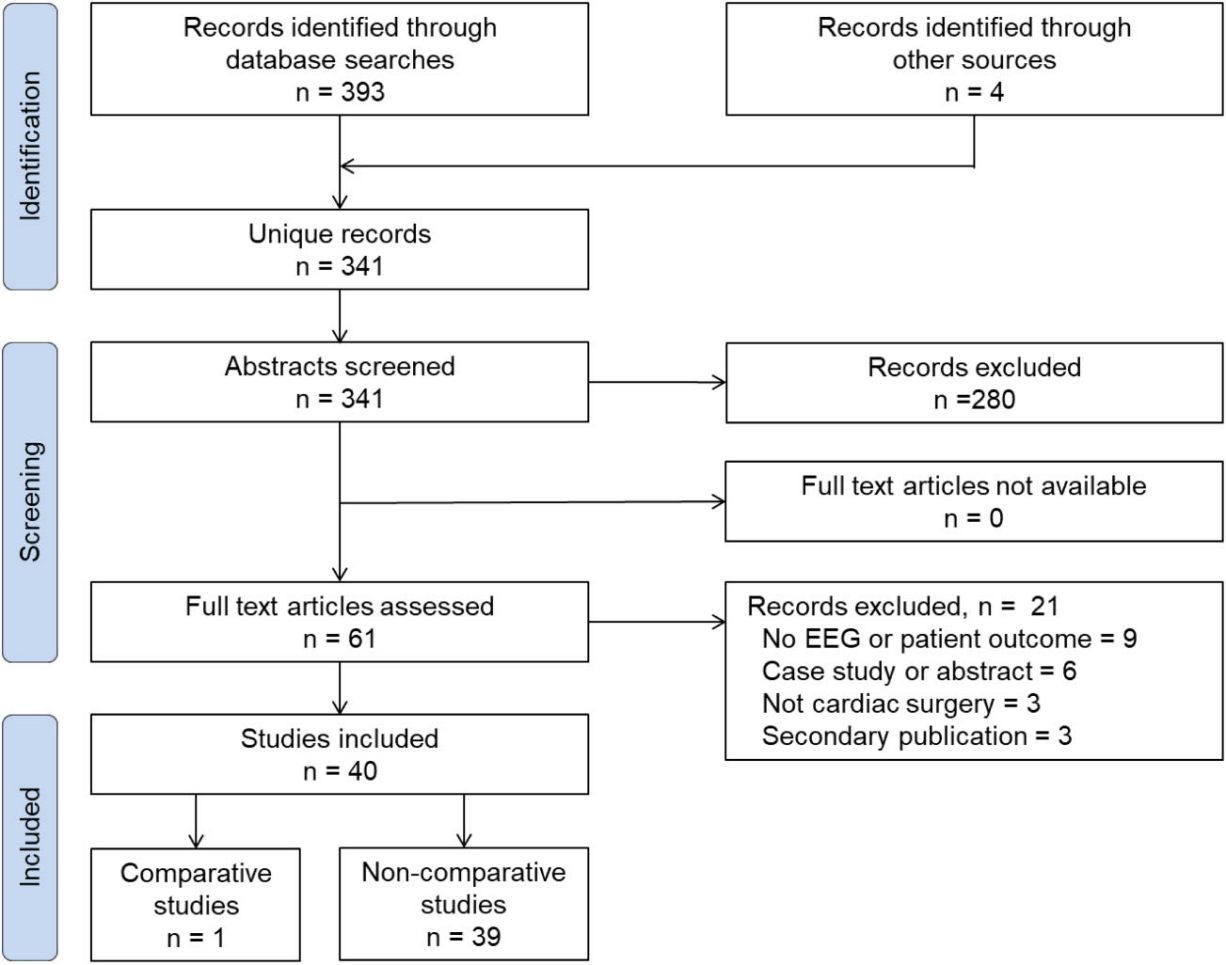
PRISMA flow diagram of study selection. EEG: electroencephalography.

From 341 unique records, we identified 40 studies with a description of perioperative EEG monitoring that included 3287 patients undergoing cardiac surgery with HCA. Only 1 study directly compared detailed outcomes between groups with perioperative EEG monitoring versus no monitoring. We therefore performed a narrative review of the non-comparative studies, and 1 comparative study, on the utility of perioperative EEG monitoring (Fig. 1).

**Figure:**
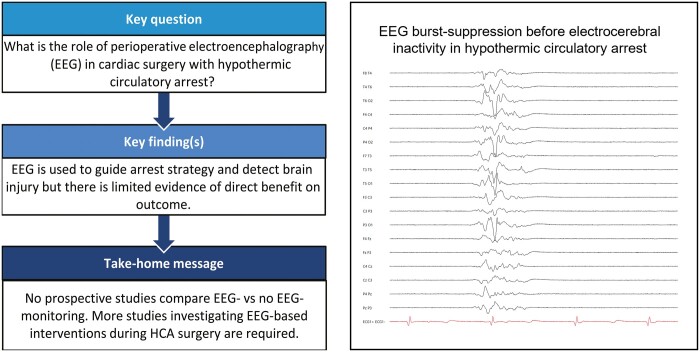


### Included studies

EEG monitoring was performed intraoperatively only (19, 48%, *n* = 2245) [11–29], postoperatively only (2, 5%, *n* = 283) [6, 30] or a combination of pre-, intra- and/or postoperatively (19, 48%, *n* = 759) [31–49]. Thirty-nine (98%, *n* = 3276) studies reported a combination of clinical and EEG outcomes of interest, the other (*n* = 11) focused solely on EEG analysis technique [27].

All studies were single centre, consisting of 6 (15%) reports of RCTs, 3 of which were sub-studies that provided additional outcome data [42, 43, 48], 18 (45%) retrospective cohort studies and 16 (40%) prospective cohort studies, 3 of which were sub-studies that provided additional data [22, 39, 47] (Table 1 and Supplementary material).

**Table 1:** Summary characteristics of included studies

Lead author	Year	Country	Study type	*n*	Age	Condition	HCA		EEG	
							Guide	End point	When used	Outcome measure/perioperative change
Algra *et al.* [46]	2014	Netherlands	RCT	37	15D (±21)	Aortic arch	Temp	18°C	Pre, intra, postop	Background, continuity, seizure/NR
Andropoulos *et al.* [40]	2010	USA	Prosp	68	8D (IQR: 5–14)	CHD	NR	NR	Pre, postop	Background, seizure/Seizure treatment
Bachet *et al.* [25]	1991	France	Retro	54	55Y (Ra: 25–76)	Aortic arch	EEG	27°C and ECI	Intra	Continuity, ECI/HCA strategy
Bavaria *et al.* [19]	2001	USA	Retro	104	58Y (±15)	Acute type A	Both	>5 mins ECI and <3°C	Intra	ECI/cannulation site
Cefarelli *et al.* [12]	2017	Netherlands	Retro	791	63Y (±11.8)	Aortic arch	Both	<18°C	Intra	Asymmetry, ECI/NR
Chen *et al.* [30]	2009	USA	Prosp	122	6D (Ra: 1–177)	CHD	Temp	18°C	Postop	Seizure/seizure treatment
Cheung *et al.* [14]	1998	USA	Prosp	18	68Y (IQR: 49–77)	Aortic arch	EEG	ECI	Intra	Continuity, ECI/HCA strategy
Drury *et al.* [31]	2013	New Zealand	Prosp	18	39M (Ra: 37–41)	TGA	Temp	22°C	Intra, postop	Amplitude, seizure/NR
Feyissa *et al.* [38]	2016	USA	Retro	32	60Y (±11.7)	Asc aorta and arch	EEG	ECI	Pre, intra	Background, ECI, seizure/NR
Ganzel *et al.* [15]	1997	USA	Retro	30	61Y (±13.3)	Mix	EEG	ECI	Intra	Continuity, ECI/HCA strategy
Gaynor *et al.* [36]	2005	USA	Prosp	183	7D (Ra: 1–188)	CHD	Temp	18°C (IQR: 15–21)	Pre, postop	Seizure/seizure treatment
Gaynor^a^*et al.* [47]	2013			132	4Y					
Ghincea *et al.* [29]	2021	USA	Retro	364	61Y (IQR: 51–68)	Aortic arch	Temp	B: 27°C (IQR: 25–28)	Intra	Asymmetry, background, frequency/HCA strategy, postop care/imaging
Hayashida *et al.* [18]	2007	Japan	Prosp	20	67Y (±9.6)	Aortic arch	Temp	N: 18°C; R: 20°C	Intra	Continuity/NR
Hirotani *et al.* [23]	2000	Japan	Prosp	75	Ra: 21–83Y	Mix	EEG	2–3°C < ECI	Intra	ECI/NR
Huang *et al.* [35]	2007	China	RCT	24	8M (IQR: 5–13)	VSD	Temp	28–30°C OR 18°C	Pre, postop	Background/NR
Iwamoto *et al.* [44]	1990	Japan	Prosp	75	6Y (±3)	CHD	NR	NR	Pre, postop	Background/NR
Jacobs *et al.* [20]	2001	Netherlands	Prosp	50	47Y (Ra: 22–70)	Asc aorta and arch	Temp	28–30°C	Intra	Asymmetry, continuity/ACP strategy
James *et al.* [17]	2014	USA	Retro	325	58Y (±14)	Prox/asc aorta, arch	EEG	ECI	Intra	Asymmetry, ECI/NR
Keenan *et al.* [13]	2016	USA	Retro	71	64Y (IQR: 53–69)	Aortic arch	Both	28°C	Intra	Continuity/ACP/HCA strategy
Ma *et al.* [11]	2020	USA	Retro	16	NR	Prox aorta and arch	EEG	ECI	Intra	Continuity, ECI/NR
Mavroudis *et al.* [32]	2018	USA	Prosp	10	4D (±1.5)	Aortic arch	Temp	18°C	Intra, postop	Amplitude, continuity, seizure/NR
Miyerbekov *et al.* [24]	1997	Russia	Prosp	9	Ra: 13–66Y	Thoracic aorta	NR	13.5°C (±0.5)	Intra	Continuity/HCA strategy
Mizrahi *et al.* [37]	1989	USA	Prosp	56	58Y (±12)	Asc aorta and arch	EEG	2°C < ECI	Pre, intra	Amplitude, continuity, ECI/HCA strategy
Murashita and Pochettino [16]	2016	USA	Retro	141	59Y (±14.6)	Aortic arch	Both	ECI	Intra	Asymmetry, continuity, ECI, seizure/HCA strategy
Naim *et al.* [6]	2015	USA	Retro	161	5D (IQR: 3–7)	CHD	Temp	NR	Postop	Seizure/seizure treatment
Newburger *et al.* [45]	1993	USA	RCT	171	10D (±11.3)	TGA	Temp	<18°C	Pre, intra, postop	Background, continuity, seizure/seizure treatment
Bellinger^b^*et al.* [43]	1995									
Helmers^b^*et al.* [48]	1996									
Helmers^b^*et al.* [42]	1997									
Raja *et al.* [49]	2003	USA	Retro	27	Ra: 9–90D	HLHS	NR	NR	Pre, postop	Background, seizure/NR
Rung *et al.* [26]	1991	USA	Retro	15	6M (±1.2)	CHD	Temp	N: 15–17°C R: 20–22°C	Intra	Amplitude, frequency, seizure/HCA strategy
Seleznev *et al.* [28]	2002	Russia	Retro	42	45Y (Ra: 14–66)	Asc aorta and arch	Temp	14–15°C	Intra	ECI/NR
Seltzer *et al.* [33]	2014	USA	Prosp	32	10D (±9.4)	CHD	Temp	21.2°C (±2.2)	Pre, intra, postop	Background, continuity, ECI, seizure/NR
Seltzer^a^*et al.* [39]	2016			21	7D (±2.4)					
Stecker *et al.* [21] and Stecker^a^*et al.* [22]	2001	USA	Prosp	109	65Y (±13.4)	Thoracic aorta	EEG	ECI	Intra	Continuity, ECI/HCA strategy
Tobochnik *et al.* [34]	2014	USA	Retro	6	64Y (IQR: 42–68)	Aortic arch	Temp	20°C	Pre, intra	Background, asymmetry, continuity, seizure/NR
Toet *et al.* [41]	2005	Netherlands	Prosp	20	8D (IQR: 6–10)	TGA	Temp	<21°C	Pre, intra, postop	Background, continuity, seizure/seizure treatment
Westover *et al.* [27]	2015	USA	Retro	11	62Y (Ra: 36–79)	Thoracic aorta	Both	18°C and ECI	Intra	Amplitude, continuity, ECI frequency/NR

a A sub-study.

b A sub-study of The Boston Circulatory Arrest Study.

ACP: antegrade cerebral perfusion; Asc: ascending; B: bladder; Both: temperature and EEG used as guide; CHD: congenital heart disease; D: days; ECI: electrocerebral inactivity; EEG: electroencephalography; HCA: hypothermic circulatory arrest; HLHS: hypoplastic left heart syndrome; Intra: intraoperative; IQR: interquartile range; M: months; Mix: mixture of heart diseases which require surgical intervention; N: nasopharyngeal; NR: not reported; Postop: postoperative; Pre: preoperative; Prosp: prospective cohort; Prox: proximal; R: rectal; Ra: range; RCT: randomized controlled trial; Retro: retrospective cohort; Temp: temperature; TGA: transposition of the great arteries; VSD: ventricular septal defect; Y: years.

Most (20, 50%) studies focused on adult patients, originated from the USA (28, 70%) and most often involved surgery to the proximal aorta or aortic arch (17, 43%). Almost all were published in English (38, 95%), most often in specialist cardiothoracic surgery journals (13, 33%). The number of participants per study ranged between 6 and 791 (median 46, interquartile range: 20–109).

### Outcome measures

The most commonly reported clinical outcome measure was postoperative neurological dysfunction, which occurred in 0–21% of patients. This broad term encompassed neurological signs and symptoms ranging from confusion to paralysis. When measuring clinical outcome, a variety of methods were used, including recognized clinical examinations (Glasgow Coma Scale, neurological examination) and imaging (ultrasound, computed tomography, magnetic resonance imaging). Outcome measures were typically assessed before hospital discharge. Only 6 (15%) studies used scales to assess long-term neuro-developmental outcome, exclusively in children [39, 41, 43, 44, 46, 47] (as shown in the Supplementary material). The majority of these studies identified that both the presence of seizures and the increasing duration of electrocerebral inactivity (ECI) on EEG were associated with poor neuro-developmental outcomes.

The most common EEG outcome measure reported was an assessment of EEG continuity during cooling and rewarming (18, 45%), which was used to predict postoperative outcome, assess anaesthetic depth and guide HCA strategy [11, 13–16, 18, 20, 22, 24, 25, 27, 32–34, 37, 41, 45, 46]. Of these, 3 (17%) studies report longer durations of non-continuous EEG following HCA associated with postoperative neurological dysfunction [11, 22, 39], and in 3, there was a trend that was either not significant or not associated with outcome [16, 25, 46]. EEG was used for seizure detection, or to predict seizure occurrence in 15 (38%) studies [6, 16, 26, 30–34, 36, 38, 40–42, 46, 49]. Seizures occurred in 0–21% of cases, of which up to 85–100% were subclinical (i.e. only detectable by EEG). The presence or duration of ECI on EEG prior to deep HCA (DHCA) was used in 15 (38%) studies as an indicator of optimized cerebral protection and to guide HCA strategy [11, 12, 14–17, 19, 21, 23, 25, 27, 28, 37–39]. Of these, 14 involved adults and 1 was exclusively in neonates [39]. With the exception of seizure monitoring, 11 (28%) studies utilized EEG to detect background abnormalities indicative of neurological injury [29, 33–35, 38, 40, 41, 44, 46, 48, 49], and the rate of EEG abnormalities detected varied between 0% and 44%.

One retrospective study directly compared clinical outcome in patients undergoing perioperative EEG monitoring against a control group [29]. They identified early detection of stroke (75% sensitivity) and accurate prediction of no stroke (97% negative predictive value) using a neuromonitoring protocol that included EEG. A single-centre study reported outcomes of aortic arch surgery using EEG-guided DHCA [16] and compared outcomes to their previously reported cohort [50]. They identified a lower rate of mortality, stroke and reoperation for bleeding in the more recent study and report the only difference between the cohorts was the use of ECI-guided DHCA.

Other infrequently reported EEG parameters included changes in the amplitude or frequency of cortical activity to detect intraoperative ischaemia in 4 (10%) studies [13, 19, 20, 29]. When detected, this resulted in a change in cannulation strategy; the depth of hypothermia prior to circulatory arrest or the CPB/cerebral perfusion pump flow/blood pressure augmentation; and postoperative imaging and catheter-based interventions. A retrospective study of aortic arch procedures identified asymmetries in EEG activity between left and right hemispheres following innominate artery cannulation, reflecting uneven active cooling of the brain [34].

The interpretation of EEG activity in relation to ECI was often defined as cortical activity <2 µV for 2–3 min. Whilst national and international guidelines for EEG interpretation are widely available [8, 51–57], few explicitly cited these standards. The number of electrodes used to record the EEG varied between 2 and 21, with 15 (38%) utilizing the full international 10–20 scalp positions [51] followed by a limited montage consisting of between 2 and 12 electrodes (13, 33%). These studies frequently cited the 10:20 system for electrode application, but few reported recording parameters such as filter settings (6, 15%) and EEG sampling rate (4, 10%). Seventeen (43%) studies provided limited or no information on the technical standards of EEG recording.

## DISCUSSION

Perioperative EEG during cardiac surgery with HCA is used to detect neurological abnormalities, seizures and ischaemia; to guide the depth of anaesthesia and hypothermia before arrest; and to predict clinical outcome. However, we found no prospective studies comparing perioperative EEG monitoring versus no EEG monitoring in adults and children undergoing cardiac surgery.

In the 40 articles included in this review, there were only 3 RCTs, none of which used EEG monitoring as the primary intervention. The remaining prospective and retrospective cohort studies demonstrated heterogeneity in the interventions used and outcome measures reported. Almost all lacked a control group of patients who were not monitored with perioperative EEG, limiting our evaluation of the role of EEG in improving postoperative outcomes. As a result, this review provides limited evidence to support EEG monitoring during HCA surgery, a technique that is used in some centres to guide surgical decision-making.

### Seizures

Perioperative seizures (which occurred in up to 21% of patients) and the cumulative burden of seizures are associated with unfavourable neurological outcomes in children [58] and adults [59] following admission to intensive care. In our review, a high proportion of seizures in the perioperative period were only detected by EEG. It would therefore seem appropriate to utilize EEG to detect these events, as recommended by guidelines for continuous EEG monitoring in neonates [52].

### Electroencephalography continuity

Monitoring EEG continuity during cooling, HCA and rewarming could indicate whether a relationship exists between the depth/rate of hypothermia, rate of rewarming and postoperative morbidity. As cooling progresses, periodic complexes intermix with background EEG activity, and the amplitude of cortical activity decreases, becoming separated by ever-increasing periods of relative electrical suppression (i.e. a burst-suppression pattern) until ECI is achieved; the reverse occurs with rewarming [21, 22, 28, 37]. The time and temperature at which these milestones occur are highly variable between patients and, thus, cannot be accurately predicted by other clinical metrics [17]. If the timing and duration of ECI are associated with neurological outcome, this would support the utility of EEG as an intraoperative neuroprotection tool to ensure that ECI has been obtained prior to DHCA.

### Electroencephalography utility

In this review, postoperative neurological dysfunction was associated with prolonged time to the return of continuous EEG activity [11, 22] and longer periods of ECI in select studies [39]. One study reviewed stroke rates in 364 adults who required aortic arch repair with HCA [29]. Of these, 223 were monitored with evoked potentials (EPs) and EEG. Surgeons were alerted when specific EP/EEG monitoring criteria were breached. Twelve developed early stroke, which was detected using EPs (9/12) and EEG (1/12). Although there were no significant differences in stroke rate between monitored and unmonitored groups, intraoperative detection of stroke in the monitored group led to earlier intervention, which may have limited brain injury. Authors provide a summary of stroke detection and intervention criteria; however, EEG-criteria breaches were infrequent, limiting the EEG monitoring evidence base.

No study reported an association between outcome and the rate and depth of cooling before DHCA. This has been shown to affect synaptic activity in animal studies, with higher rates of cooling causing a progressive decrease in activity and lower rates promoting the preservation of activity and tissue plasticity [60]. Fast rewarming can cause brain injury in both animal [61] and human studies [62]. This may explain some of the variation seen in the time to achieve ECI and return of continuous EEG activity, although many other factors including anaesthetic regime may contribute to this finding.

As moderate hypothermia is being slowly introduced in surgical practice, the role of EEG could move from ensuring ECI prior to circulatory arrest to preserving some degree of continuity in the EEG. In a recent series, EEG monitoring was used during hemiarch replacement with moderate HCA and selective antegrade cerebral perfusion (SACP) [13]. Immediately after circulatory arrest, ECI occurred in 45% of patients, which was indicative of cerebral ischaemia. EEG activity was re-established following SACP in all but 2 cases; in one, asymmetric activity was restored following bilateral ACP, and in the other, CPB was re-established and the depth of hypothermia increased before circulatory arrest. They concluded that intraoperative EEG may have specific value in identifying patients with persistent cerebral ischaemia, even after SACP.

### Role of electroencephalography in perioperative monitoring

The EACTS/ESVS 2019 expert consensus document on the management of thoracic aortic and aortic arch disease identified widespread use of perioperative EEG monitoring but a lack of evidence for an incremental benefit [63]. Similarly, a systematic review and meta-analysis on outcomes in children following DHCA with EEG monitoring identified 19 studies published in English since 1990 [64]. They reported similar pooled event rates of clinical seizures (12.9%), EEG seizures (14.9%), neurological abnormalities (29.8%) and EEG abnormalities (17.3%) to our findings in adults and children. They concluded that despite its frequent use EEG remains poorly studied.

To facilitate the synthesis of findings from multiple studies, valid and comparable outcome measures must be reported [65]. We found variation in the utilization, acquisition and recording period of EEG, with inconsistent use of measures to evaluate the same outcome. EEG continuity, seizure and ECI monitoring were the most commonly used metrics but not all mention how EEG patterns and seizures were classified. Similarly, metrics of neurological dysfunction were the most commonly reported clinical outcome measure, but signs and symptoms used to define this were broad, ranging from confusion to paralysis, and measurements were performed at variable time points. This disparity reflects the absence of a standardized method for reporting perioperative EEG and the measurement of clinical outcomes following cardiac surgery.

Long-term assessment of neurological outcomes following surgery with HCA represents the gold standard to detect more subtle yet persistent neurological deficits, but these were infrequently performed, perhaps due to the burden it places on participants and researchers. In addition, those who require surgery with HCA typically have a multitude of pre-existing comorbidities, heterogeneous and complex heart disease and variability in the length of postoperative hospital stay; measures used to define neurological outcomes during early childhood are also dependent on age. These factors make it inherently difficult to attribute postoperative injury to any one factor and a challenge to compare the neonatal, child and adult populations. Other than for the detection of postoperative seizures, there is currently limited evidence supporting EEG metrics, such as disappearance/return of EEG continuity, or the time and duration of ECI in guiding perioperative care. In studies that monitored ECI attainment before DHCA, a consistent definition of ECI, and how long it needs to be established before circulatory arrest is required.

### Limitations

The limitations of this review include the lack of RCTs directly comparing the use of EEG monitoring with a control group; our inability to perform a meta-analysis due to the lack of comparable outcome measures; and a risk of reporting bias, although minimized by performing an extensive search across multiple databases and including non-English language articles.

## CONCLUSIONS

Perioperative EEG monitoring in cardiac surgery with HCA can detect seizures and neurological abnormalities, identify intraoperative ischaemia and may guide cerebral protection and predict outcome. However, the inconsistent metrics used to record, acquire and interpret EEG, and clinical outcome measures limit the evidence base to inform clinical practice. No prospective studies compared perioperative EEG versus no perioperative EEG monitoring, and thus, there is a lack of direct evidence to demonstrate whether EEG monitoring may have a role in improving clinical outcomes. An assessment of EEG continuity during HCA could provide insight into improving perioperative cerebral protection. A standardized approach to EEG monitoring during HCA and postoperative clinical outcome reporting is required to inform the design of future clinical trials.

## SUPPLEMENTARY MATERIAL

Supplementary material is available at *ICVTS* online.

## Supplementary Material

ivac198_Supplementary_Data

## Data Availability

The data underlying this article are available in the article and in its online supplementary material.

## Notes

Presented at the virtual Society for Cardiothoracic Surgery in Great Britain and Ireland National Research Meeting, 4 November 2021, Virtual Presentation.

## ABBREVIATIONS

CPB: Cardiopulmonary bypass
DHCA: Deep hypothermic circulatory arrest
ECI: Electrocerebral inactivity
EEG: Electroencephalography
EPs: Evoked potentials
HCA: Hypothermic circulatory arrest
RCT: Randomized controlled trial
SACP: Selective antegrade cerebral perfusion
