# Supplementary material for: Perioperative electroencephalography in cardiac surgery with hypothermic circulatory arrest: a narrative review
Source: Interact Cardiovasc Thorac Surg. 2022 Jul 29;35(4):ivac198. doi: 10.1093/icvts/ivac198 (PMC9462422; doi:10.1093/icvts/ivac198)
Supplement: ivac198_Supplementary_Data [file ivac198_supplementary_data.docx]

**SUPPLEMENTARY MATERIAL**

**Data Items and descriptors**

Study descriptors

Surname of first author (text)
Article published in (text)
Year of publication (numerical)
PubMed Identifier (PMID) (numerical)
Number of secondary publications (numerical)
Identified in which search engine (categorical)
Language published in (text)

Study demographics & design

Number of centres at which patients underwent surgery (numerical)
Name(s) of centres at which patients underwent surgery (text)
Number of countries in which patients were recruited (numerical)
Name(s) of countries in which patients were recruited (text)
Type of study (categorical)
Number of participants included in analysis of the primary outcome (numerical)
Age (categorical)
Condition (text)
Study objective (text)
Intervention (text)
Control (categorical)
Intervention details (text)
HCA guide (categorical)
Temperature probes used (text)
Temperature used to perform HCA (numerical)
Duration of cardiopulmonary bypass, cross clamp, HCA, ACP (numerical)
Anaesthetic regime (text)

Outcomes

EEG implementation (categorical)
Number and location of EEG electrodes applied (text)
Were EEG application guidelines used (categorical)
Was polygraphy used (text)
Sampling rate and EEG filter settings (text)
EEG outcome measure (text)
ECI interpretation guidelines (text)
EEG interpretation guidelines (text)
Duration of EEG monitoring (numerical)
EEG success rate (numerical)
Duration of cooling, rewarming, ECI (numerical)
Time to ECI from cooling initiation; recover EEG activity/burst suppression, continuity (numerical)
Temp Burst suppression, ECI achieved (numerical)
EEG change during cerebral perfusion (text)
Asymmetry seen in EEG (text)
Interpretation of EEG (categorical)
Change in operation of postop care due to EEG change (text)
How many had seizures (numerical)
Type of primary outcome, e.g. clinical, EEG, other (categorical)
Other outcomes reported (text)
Main finding related to EEG (text)

**Search Strategy**

MEDLINE

Date run: 10^th^ May 2022

#1 (hypothermic and circulatory and (assist or arrest)).mp. [mp=title, abstract, original title, name of substance word, subject heading word, floating sub-heading word, keyword heading word, organism supplementary concept word, protocol supplementary concept word, rare disease supplementary concept word, unique identifier, synonyms] (3086)

#2 induced hypothermia.mp. or exp Hypothermia, Induced/ (23337)

#3 (medical hypothermia or therapeutic hypothermia or protective hypothermia or medical cooling).mp. [mp=title, abstract, original title, name of substance word, subject heading word, floating sub-heading word, keyword heading word, organism supplementary concept word, protocol supplementary concept word, rare disease supplementary concept word, unique identifier, synonyms] (4352)

#4 1 or 2 or 3 (26788)

#5 Electroencephalography/ (156610)

#6 electroencephalography.mp. [mp=title, abstract, original title, name of substance word, subject heading word, floating sub-heading word, keyword heading word, organism supplementary concept word, protocol supplementary concept word, rare disease supplementary concept word, unique identifier, synonyms] (165032)

#7 5 or 6 (165032)

#8 4 and 7 (1031)

#9 exp Perioperative Nursing/ or exp Perioperative Care/ or exp Perioperative Period/ (258202)

#10 exp Intraoperative Care/ or exp Intraoperative Period/ or exp Intraoperative Neurophysiological Monitoring/ or exp Intraoperative Complications/ or exp Monitoring, Intraoperative/ (117225)

#11 exp Preoperative Period/ or exp Preoperative Care/ (81549)

#12 exp Postoperative Period/ or exp Postoperative Care/ or exp Postoperative Complications/ (687964)

#13 (perioperative or postoperative or intraoperative or preoperative).mp. [mp=title, abstract, original title, name of substance word, subject heading word, floating sub-heading word, keyword heading word, organism supplementary concept word, protocol supplementary concept word, rare disease supplementary concept word, unique identifier, synonyms] (1183624)

#14 exp Thoracic Surgery/ (13367)

#15 ((heart or cardiac or cardiothoracic) and (surgery or surgical)).mp. [mp=title, abstract, original title, name of substance word, subject heading word, floating sub-heading word, keyword heading word, organism supplementary concept word, protocol supplementary concept word, rare disease supplementary concept word, unique identifier, synonyms] (335672)

#16 9 or 10 or 11 or 12 or 13 or 14 or 15 (1541018)

#17 8 and 16 (239)

#18 limit 17 to yr="2021 -Current" (4)

EMBASE

Date run: 10^th^ May 2022

#1 induced hypothermia.mp. or exp induced hypothermia/ (17678)

#2 (hypothermic and circulatory and (assist or arrest)).mp. [mp=title, abstract, heading word, drug trade name, original title, device manufacturer, drug manufacturer, device trade name, keyword heading word, floating subheading word, candidate term word] (4907)

#3 (medical hypothermia or therapeutic hypothermia or protective hypothermia or medical cooling).mp. [mp=title, abstract, heading word, drug trade name, original title, device manufacturer, drug manufacturer, device trade name, keyword heading word, floating subheading word, candidate term word] (7454)

#4 1 or 2 or 3 (22973)

#5 electroencephalography.mp. or exp electroencephalography/ (136790)

#6 4 and 5 (804)

#7 exp perioperative period/ or exp perioperative medicine/ (60994)

#8 exp intraoperative period/ or exp intraoperative monitoring/ (229817)

#9 exp postoperative care/ or exp postoperative complication/ or exp postoperative period/ (1201670)

#10 exp preoperative period/ or exp preoperative care/ or exp preoperative evaluation/ (367861)

#11 (perioperative or postoperative or intraoperative or preoperative).mp. [mp=title, abstract, heading word, drug trade name, original title, device manufacturer, drug manufacturer, device trade name, keyword heading word, floating subheading word, candidate term word] (1561996)

#12 ((heart or cardiac or cardiothoracic) and (surgery or surgical)).mp. [mp=title, abstract, heading word, drug trade name, original title, device manufacturer, drug manufacturer, device trade name, keyword heading word, floating subheading word, candidate term word] (565846)

#13 exp heart surgery/ (408993)

#14 7 or 8 or 9 or 10 or 11 or 12 or 13 (2532159)

#15 6 and 14 (190)

#16 limit 15 to yr="2021 - 2022" (15)

CENTRAL

Date run: 10^th^ May

#1 MeSH descriptor: [Electroencephalography] explode all trees (5307)

#2 electroencephalography OR EEG (11734)

#3 #1 OR #2 (12129)

#4 hypothermic circulatory AND (assist OR arrest) (140)

#5 medical hypothermia OR therapeutic hypothermia OR protective hypothermia (1977)

#6 induced hypothermia (1839)

#7 medical cooling (634)

#8 #4 OR #5 OR #6 OR #7 (3320)

#9 #3 AND #8 (124)

#10 MeSH descriptor: [Perioperative Care] explode all trees (12780)

#11 MeSH descriptor: [Perioperative Period] explode all trees (9235)

#12 MeSH descriptor: [Perioperative Nursing] explode all trees (129)

#13 MeSH descriptor: [Preoperative Care] explode all trees (6133)

#14 MeSH descriptor: [Postoperative Care] explode all trees (4615)

#15 perioperative OR postoperative OR intraoperative OR preoperative (162467)

#16 MeSH descriptor: [Thoracic Surgery] explode all trees (176)

#17 (cardiac OR heart) AND (surgery OR surgical) (45672)

#18 MeSH descriptor: [Intraoperative Period] explode all trees (2798)

#19 #10 or #11 OR #12 or #13 or #14 OR #15 OR #16 OR #17 OR #18 (187817)

#20 #9 AND #19 (31)

#21 limit 20 to yr=“1985 -Current” (31)

#22 #9 AND #19 with Cochrane Library publication date Between Jan 2021 and May 2022 (1)

LILACS

Date run: 11 March 2021

#1 tw:(electroencephalo* OR electroencefalo*)

#2 tw:(hypotherm* OR hipotermia)

#3 #1 AND #2 (8)

Date run: 13th May 2022

#1 tw:(electroencephalo* OR electroencefalo*)

#2 tw:(hypotherm* OR hipotermia)

#3 #1 AND #2 (8)

| **Lead Author, year** | **Time/Temp**  **ECI achieved** | **Time/ Temp to continuous EEG** | **Neurological outcome measure/**  **scale** | **EEG injury**  **measure, rate** | **Comment/Notes** |
| --- | --- | --- | --- | --- | --- |
| Algra, 2014 | NR | DHCA: 5.8 hours;  ACP: 1.7 hours | Neurological dysfunction, MRI, NIRS, NSE, s100b/Bayley-III | Seizures, 19% | Majority of seizures were subclinical; EEG continuity took longer to achieve in DHCA group. |
| Andropoulos, 2010 | NR | NR | NIRS, MRI,  neurological dysfunction | Seizures,  0.7% | Preop EEG 100% normal; Seizures correlated with MRI lesion; not all EEG abnormalities associated with outcome |
| Bachet, 1991 | Mean:9 mins  (R: 3-16) | Mean:  66 mins | Mortality, Neurological, respiratory and renal dysfunction | NR | More likely to have major postop complications the longer EEG took to become continuous postop |
| Bavaria, 2001 | 29 mins (±17)/  R:15-20°C | NR | Mortality,  Neurological dysfunction | NR | EEG best tool to guide HCA and may have prevented stroke |
| Cefarelli, 2017 | NR | NR | NIRS, TCD, Mortality, Neurological dysfunction, CT, MRI | NR | TCD preoperatively to identify CoW abnormalities;  vast majority required bilateral CP |
| Chen, 2009 | NR | NR | MRI,  Neurological dysfunction | Seizures,  1% | Seizures correlated with  MRI lesion |
| Cheung, 1998 | 25 mins (±13)/ 17.7°C (±3.9) | 48 mins  (R: 20-108) | Neurological dysfunction | Seizures,  6% | Time to EEG reappearance weakly associated with nasopharyngeal temp |
| Drury, 2013 | NR | NR | MRI & NIRS | Seizures,  17% | Three infants (2 in DHCA group, 1 in non-DHCA group) had  electrographic seizures during CPB |
| Feyissa, 2016 | 23.1°C  (±4) | 33.8°C  (±2.3) | Mortality, Neurological dysfunction/  CPC Score | Asym post HCA, 9% | No correlation between EEG patterns and outcome |
| Ganzel, 1997 | 17°C (±3.7); R: 8-22 | RCP: 21 mins (±12)  No RCP: 55 (±49) | NIRS, Neurological dysfunction,  mortality | NR | Temp at which ECI occurs is variable and activity returns quicker if you use cerebral perfusion |
| Gaynor, 2005 | NR | NR | MRI, Neurological dysfunction | Seizures, 11% | All seizures were subclinical |
| Gaynor, 2013* |  |  | Full-Scale IQ, Preschool Language Test-4 Total Language Score, Wide Range Assessment of Visual Motor Abilities pegboard, Developmental Test of Visual Motor Integration, reading and math clusters of the Woodcock-Johnson III, NEPSY score, ADHD scale-IV preschool version, Preschool and Kindergarten Behavior Rating Scales Social Skills Total Score, The Child Behavior Checklist |  | greater duration of DHCA with history of seizures; patients with a history of seizure had worse performance for executive function; occurrence of a seizure after cardiac operation is a biomarker of CNS injury; outcome tests performed <1 years of age might not identify associations between outcome and EEG findings |
| Ghincea, 2021 | NR | NR | Neurological dysfunction, CT, MRI | Slowing, 11% | SSEP, MEP better diagnostic accuracy than EEG for stroke detection |
| Hayashida, 2007 | R: 16-24°C | NR | Neurological dysfunction | NR | Rate of BIS recovery related to duration of DHCA. |
| Hirotani, 2000 | NR | NR | CT, Neurological dysfunction,  Mortality | NR | Pharmacological brain protection reported as effective for safely extending hypothermic circulatory arrest |
| Huang, 2007 | NR | NR | NIRS, Blood NSE & lactate, Neurological dysfunction, Mortality | NR | abnormal postop EEGs had longer HbO2-signal nadir time compared to those with normal EEGs |
| Iwamoto, 1990 | NR | NR | Neurodevelopment/  IQ test | NR | 16% had abnormal preop EEG, 17% abnormal post op but this did not predict outcome |
| Jacobs, 2001 | NR | NR | Mortality, CT, Neurological &  Renal dysfunction | NR | EEG missed surgery-acquired brain injury |
| James, 2014 | 69 mins (±17)/  15.5°C (±1.9) | NR | Neurological dysfunction,  Renal dysfunction | NR | Cooling to 12.7°C required to attain ECI in all patients |
| Keenan, 2016 | NR | NR | Neurological dysfunction | ECI post HCA, 45%  Asym post uSACP, 3% | Use of EEG in moderate HCA |
| Ma, 2020 | NR | 74 mins (±31.4); R: 22-239 | Neurological dysfunction (Delirium)/ RASS &CAM-ICU | NR | Warming earliest opportunity to predict postop delirium via EEG |
| Mavroudis, 2018 | 20.2°C | 35.5°C (±0.3) | MRI | NR | Majority did not achieve ECI pre DHCA, and only one had postop injury |
| Mierbekov, 1997 | 13.5°C  (±0.5) | NR | Mortality,  Neurological dysfunction | NR | Cooling doesn’t stop metabolic activity, even at 13.5°C |
| Mizrahi, 1989 | 41 mins (R: 20-107) | NR | Survival,  Neurological dysfunction | NR | Variable temp at which ECI occurred |
| Murashita, 2016 | NR | NR | Neurological dysfunction, prolonged ventilation | Asym, suppression, seizure post HCA, 4.3% | EEG detected stroke but also missed it; asymmetries did not always manifest as postop deficit |
| Naim, 2015 | NR | NR | US & MRI | Seizures, 8% | 85% of seizures were non-convulsive |
| Newburger, 1993 | NR | NR | Blood serum analysis, Neurological dysfunction/Neurologic exam | Seizures,  19% | DHCA Vs LfCPB, Seizures were common |
| Bellinger^¥^,   1995 |  |  | MRI/Fagan test, Bayley DS |  | Circulatory arrest is associated with a higher risk of neurologic   dysfunction than surgery with low-flow bypass |
| Helmers^¥^,   1996 |  |  |  |  | Majority of seizures subclinical, with fronto-central onset, occurring   between 13-36 hours postop; >1000 seizures recorded |
| Helmers [2]^¥^,   1997 |  |  |  |  | 35% have abnormal preop EEG;  Seizures occurred between 13-36 hours postop |
| Raja, 2003 | NR | NR | Neurological dysfunction, CT, MRI | Seizures, 21% | Abnormal preop EEG correlated with the likelihood of ongoing post op antiepileptic drug treatment. Preop EEG abnormalities (23%); DHCA time was inversely proportional to occurrence of seizures |
| Rung, 1991 | 17.8 °C  (±2) | NR | Neurological dysfunction | Seizures,  13% | Variability in when ECI is achieved,  thiopental considered pre-HCA |
| Seleznev, 2002 | 15-17°C | NR | Neurological dysfunction | NR | DHCA <17 |
| Seltzer [1], 2014 | 21.2°C (± 2.2) | NR | US, Seizures | Abn EEG post DHCA, 12%; | 10% had abnormal preop EEG; all EEGs developed ECI <25°C |
| Seltzer [2]*,   2016 |  |  | Neurodevelopment/  Vineland-II score | Seizures, 9% | Increased ECI duration associated with worse outcome |
| Stecker [1], 2001  Stecker [2],   2001* | 27.5 mins (±10); R: 12-50  17.8°C (±4)   R:12.5-27.2 | 80.5 mins (±28); 36.2°C (±0.8) Abn  47.1 mins (±26);   30.1°C (±5) norm | Neurological dysfunction, hospital discharge, intubation duration, CT | NR | Prolonged time/temp to return of continuous EEG activity is associated with postop neuro impairment  60% of patients demonstrated ECI and cooling rate correlated with   time to ECI |
| Tobochnik, 2014 | NR | NR | Neurological dysfunction | Asymmetry, 100%  Seizures, 17% | Nb: Asymmetry not a metric for injury- relates to uneven brain cooling; innominate artery cannulation caused asymmetry in all EEGs |
| Toet, 2005 | 19.3°C  (R: 17-25) | R: 8 hours -  several days | NIRS, US, MRI, Neurodevelopmental outcome/ Griffiths MDS, Bayley DS | Seizures,  5% | Preop EEG always normal;  some EEGs took days to recover |
| Westover, 2015 | 22.6°C  (± 2.8) | NR | NR | NR | Cooling to 18°C did not always cause ECI and ECI was not maintained for prolonged periods |

**Table S1**: Additional characteristics of included studies. ¥ indicates a sub-study of The Boston Circulatory Arrest Study; *Indicates a sub-study
